# Patient Perspectives of Inpatient Telemedicine During the COVID-19 Pandemic: Qualitative Assessment

**DOI:** 10.2196/32933

**Published:** 2022-03-30

**Authors:** Stacie Vilendrer, Sarah Sackeyfio, Eliel Akinbami, Roy Ghosh, Jacklyn Ha Luu, Divya Pathak, Masahiro Shimada, Emmanuelle Elise Williamson, Lisa Shieh

**Affiliations:** 1 Division of Primary Care and Population Health Stanford University School of Medicine Stanford, CA United States; 2 Department of Physics Stanford University Stanford, CA United States; 3 Department of Bioengineering Stanford University Stanford, CA United States; 4 Department of Bioinformatics Stanford University School of Medicine Stanford, CA United States; 5 Department of Biochemistry Stanford University Stanford, CA United States; 6 Department of Health Research and Policy Stanford University School of Medicine Stanford, CA United States; 7 Department of Engineering Stanford University Stanford, CA United States; 8 Division of Hospital Medicine Stanford University School of Medicine Stanford, CA United States

**Keywords:** telemedicine, inpatient, patient experience, COVID-19, infection control, quality of health care, communication, hospital, perspective, qualitative

## Abstract

**Background:**

Telemedicine has been adopted in the inpatient setting to facilitate clinical interactions between on-site clinicians and isolated hospitalized patients. Such remote interactions have the potential to reduce pathogen exposure and use of personal protective equipment but may also pose new safety concerns given prior evidence that isolated patients can receive suboptimal care. Formal evaluations of the use and practical acceptance of inpatient telemedicine among hospitalized patients are lacking.

**Objective:**

We aimed to evaluate the experience of patients hospitalized for COVID-19 with inpatient telemedicine introduced as an infection control measure during the pandemic.

**Methods:**

We conducted a qualitative evaluation in a COVID-19 designated non–intensive care hospital unit at a large academic health center (Stanford Health Care) from October 2020 through January 2021. Semistructured qualitative interviews focused on patient experience, impact on quality of care, communication, and mental health. Purposive sampling was used to recruit participants representing diversity across varying demographics until thematic saturation was reached. Interview transcripts were qualitatively analyzed using an inductive-deductive approach.

**Results:**

Interviews with 20 hospitalized patients suggested that nonemergency clinical care and bridging to in-person care comprised the majority of inpatient telemedicine use. Nurses were reported to enter the room and call on the tablet far more frequently than physicians, who typically entered the room at least daily. Patients reported broad acceptance of the technology, citing improved convenience and reduced anxiety, but preferred in-person care where possible. Quality of care was believed to be similar to in-person care with the exception of a few patients who wanted more frequent in-person examinations. Ongoing challenges included low audio volume, shifting tablet location, and inconsistent verbal introductions from the clinical team.

**Conclusions:**

Patient experiences with inpatient telemedicine were largely favorable. Although most patients expressed a preference for in-person care, telemedicine was acceptable given the circumstances associated with the COVID-19 pandemic. Improvements in technical and care team use may enhance acceptability. Further evaluation is needed to understand the impact of inpatient telemedicine and the optimal balance between in-person and virtual care in the hospital setting.

## Introduction

The COVID-19 pandemic created an unprecedented challenge for health systems to provide high-quality care to potentially contagious patients while simultaneously keeping their workforce and uninfected patients safe [1]. In response, telemedicine was widely adopted worldwide in outpatient settings [2]. Some further adopted “inpatient telemedicine” in acute care settings to facilitate clinical interactions between on-site clinicians and isolated inpatients for the purpose of infection control and to reduce the use of personal protective equipment (PPE) [3-7]. Early evidence points to the feasibility of inpatient telemedicine as an infection control measure [3-5], but formal evaluations into their use and practical acceptance among stakeholders, particularly patients, are limited.

In the outpatient setting, robust literature suggests overall patient satisfaction with telemedicine, including in general medicine [8], urgent care [9], and specialized settings [10-12]. Factors driving increased patient satisfaction include improved outcomes, decreased travel time, and improved communication [13]. Adequate training of the staff providing telemedicine services can also improve patient satisfaction [14]. Patient acceptance of telemedicine has persisted through the pandemic [15-18], with the highest levels of adoption observed among young patients [19]. However, high rates of patient satisfaction with telemedicine in the outpatient setting do not necessarily translate to the inpatient setting, given significant differences in the clinical environment, acuity of disease, frequency of clinical interactions, and the multidisciplinary team required to care for the patient.

Prior to the COVID-19 pandemic, the use of telemedicine in inpatient settings was limited to connecting rural areas with remote expertise [20,21], particularly specialty care [14,22-24]. The COVID-19 pandemic removed traditional barriers to telemedicine adoption, including staff resistance to change, and lack of reimbursement [13]. Since the pandemic, hospitalists [3,5], intensivists [4,6], and specialists [25,26] have used inpatient telemedicine to continue their clinical duties while minimizing pathogen spread. This novel use of inpatient telemedicine is still being explored: in one academic medical setting, the technology was broadly accepted by clinicians and staff but required nurses to drastically alter their workflow practices [27]. In another setting, in-room video technology saw the greatest adoption among patients who used it to communicate with their loved ones [28]. Little else is formally known about how patients perceive inpatient telemedicine, particularly under isolation conditions that a COVID-19 diagnosis necessitates.

This is unfortunate, as the patient perspective is integral to ensuring robust patient–provider communication, the lack of which can propagate diagnostic errors and adverse patient outcomes [29-31]. Isolated patients may be more likely to receive substandard care, particularly related to patient safety [32] and staff responsiveness to time-sensitive needs [33,34]. Further, patients hospitalized under isolation precautions face unique challenges, including negative feelings associated with loneliness, stigma, and fear [31-37]. Incorporating patient insights into the design of technological solutions within the patient environment is needed to support patient autonomy, human connection, and a sense familiarity that is otherwise lost during isolation [36-38].

We therefore aim to understand patient experience as it relates to clinical use and patient acceptance of inpatient telemedicine for infection control in order to optimize technical setup and clinical workflows.

## Methods

### Methods Overview

We conducted a qualitative evaluation of inpatient telemedicine use on a COVID-19 designated inpatient unit at a large academic medical center. We analyzed qualitative interviews with stable patients hospitalized for COVID-19 to understand the implementation outcomes related to the practical use and acceptance of the inpatient telemedicine solution from the patients’ perspective.

### Setting and Population

In March 2020, a large academic health center (Stanford Health Care) designated an inpatient non–intensive care unit to care for admitted patients with confirmed or suspected COVID-19. Each patient received a tablet (iPad; Apple Inc) set up to automatically receive web conference calls (Zoom Video Communications, Inc) from one of two desktops in private workrooms on the unit, all with dual audio and video functionality. Other setting details have been previously described [3] and can be found in Multimedia Appendix 1.

The target population included stable patients who were admitted to the non–intensive care COVID-19 unit. Purposive sampling methods were used to recruit participants representing diversity across age, sex, race and ethnicity, and language until thematic saturation was reached [39].

### Data Collection and Analysis

The semistructured patient interview protocol, analysis, and presentation of findings were informed through deductive themes drawn from prior qualitative work [27] and the Implementation Outcomes Framework (Multimedia Appendix 1) [40]. Interview transcripts were reviewed independently by the lead author (SV), and key themes and inductive coding that emerged were then validated using a consensus approach that included the analytic (DP, MS, JHL, and RG) and interview (SS, EA, and EEW) teams.

### Ethical Considerations and Informed Consent

The Stanford Institutional Review Board determined that this project did not qualify as human subjects research (protocol 55927). All participants provided verbal consent to proceed and were informed their participation was voluntary and confidential.

## Results

### Participant Characteristics

A total of 20 interviews were conducted with inpatients undergoing treatment for COVID-19 without intensive care needs. Participants tended to be male (11/20, 55%); aged 50-69 years (15/20, 75%); Latino, White, or Asian, Indian, or Pacific Islander (6/20, 30% each), and spoke primarily English (16/20, 80%) (Multimedia Appendix 1).

### Reported Use and Acceptance of Inpatient Telemedicine From the Patients’ Perspective

Telemedicine use varied on the basis of the clinical context and the type of clinician providing care, though it was broadly accepted by patients given the COVID-19–related isolation precautions (Table 1). Predominant use included nonemergency clinical care and bridging to in-person care—such as when a patient triggered the bedside alert button. In this setting, one nurse was reported to initiate a telemedicine encounter to visually connect with the patient while another donned PPE to evaluate the patient in person.

Typical frequency of virtual encounters ranged from 1 to 3 times a day, though one patient reported being contacted up to 10 times per day via the tablet device and 10 times per day in person. Nurses were reported to both enter the room and call on the tablet more frequently than physicians. However, most patients reported at least one in-person encounter daily from their doctor, with a few reporting multiple in-person encounters when their needs were greater. A few patients reported their physician primarily communicated via the tablet device, and a minority perceived there were some days on which they did not speak with their doctor either in person or via the tablet device.

Patients generally accepted the use of inpatient telemedicine given their circumstances requiring isolation precautions. A desire to prevent infection transmission was recognized by several patients: “It’s helpful that I can talk to multiple doctors without having to expose them to COVID” [Patient 9]. Some felt that telemedicine offered superior convenience, and the visual component was seen as adding value beyond an audio-only telephone encounter. The virtual interactions provided reassurance, which was reported to positively impact patient mood and mental health: “I love to hear from my doctors, it brought such great comfort for them to be updating me” [Patient 6].

Patients broadly reported that the quality of care they received with the integration of telemedicine into their hospitalization was similar to what they might have received without it.

However, some were in favor of an in-person encounter in accordance with the concern that some aspects of their care could be missed, particularly related to the in-person examination and human connection. Some felt that telemedicine changed the way they communicated with their care team: “In person is better…I can have a more in-depth dialogue, ask questions…When they’re physically here, they can be there for me” [Patient 18]. Older patients, in particular, seemed to prefer in-person encounters. Only a handful of patients felt telemedicine did not compromise their connection with their care team.

**Table 1:** Patient perspectives on the use and acceptance of inpatient telemedicine in the context of the COVID-19 pandemic.

Learnings			Example quotations
**Reported use of inpatient telemedicine**			
	Used primarily for nonemergency care and as a bridge to in-person care	“We run the visits based on my bladder which is about every hour and a half. They [nurses] bring breakfast and then I pee and then they do my meds and stuff. Normally they don’t come in first, [instead] they check on me on [the tablet] and then come in.” [Patient 20]	
	Nurses reported to both enter the room and call on the tablet device	“Five or six times a day [in-person nurse visits] and they [nurses] also call on the iPad…” [Patient 9]	
	Physicians sometimes used telemedicine to replace in-person encounters	“At least once maybe twice a day [in-person physician encounter]. There were some days though where I didn’t have any doctors come in and talk to me. They were just conversing with the staff regarding my wellbeing.” [Patient 6]	
**Acceptance of inpatient telemedicine**			
	Generally accepted given the circumstances surrounding the COVID-19 pandemic	“Of course there’s nothing more effective than the person to person contact, but this is the safest, and the tech is so advanced it’s like you’re right in front of me” [Patient 15]	
	Telemedicine seen as improving convenience	“I think it's great because we have more contact with [the] medical providers than any other time. You normally have to wait till they have time to come and talk to you…[but this way] seems to be a lot more efficient way to handle things” [Patient 2]	
	Immediate accessibility to clinical team via telemedicine offered reassurance	“On the first day I went to sleep, woke up with anxiety real bad, feeling as though I was on my last breath. I pushed the [bedside alert] button and the nurses came on and then after that the [tablet] did come on they were looking at me, talking to me, and helped me calm down right away. They were like, ‘Just breathe, someone’s coming right in’.” [Patient 16]	
	Visual component seen as adding value over a telephone call	“…they [clinicians] are right there when I need them and I can physically see them versus waiting to talk to them via the phone.” [Patient 8]	
	No perceived impact on quality of care overall with a minority concerned about the reduction in in-person examinations	“Honestly I don’t think it has changed the care because the nurse is always reporting to the doctor and then the doctor looks at all my labs and things so they are pretty on top of it and then they call me to let me know the plan…But I wouldn’t say it’s better it’s like the same.” [Patient 1] “Most of time when we see doctors normally, they use the stethoscope, to listen to lungs, heart…That’s the normal way to see a patient, so I just wonder, I’m completely okay with the [tablet], but somehow the doctor can’t see [me] in person…the nurse never uses the [stethoscope] to listen to my lungs/heart. So that is something I am not comfortable with.” [Patient 4]	
	Some reported loss of human connection	“…on the internet you can say whatever you want; telemedicine is close to internet separation…the non-word ways of communication are much different than in person, tone, intonation…like I’m here but I don’t see your whole body and vice versa. [It] changes [one’s] mood...” [Patient 3]	

### Technical Factors Impacting Patient Acceptance

In general, the technology was reportedly “very simple to use” [Patient 5]. However, certain technical setup considerations impacted patient acceptability, including audio volume, tablet position both within the room and in relation to the patient, and the automatic turn-on feature. Several patients remarked that despite turning the tablet volume up to its maximum capacity, they still had difficulty hearing the clinical team, particularly if someone was speaking further away from the source microphone.

Furthermore, the tablet location and angle relative to the patient sometimes posed challenges. The tablet device was reportedly moved frequently to allow space for nurses doing their clinical work. This lack of a stable location worsened the experience for some patients:

Mostly I don’t like it. I don’t understand them sometimes. Sometimes they move it towards my feet. It’s too far from me, it should be near my face...

Patient 19

There was not a clear consensus on where the tablet should be placed within the room, as another patient preferred it near the feet to optimize the visual component of the encounter.

Finally, patients were asked to comment on the automatic turn-on feature of the tablet device, which did not allow them to screen calls. Nearly all were comfortable with this feature, likening it to standard hospital care in which a clinician would simply walk into the room, only sometimes following a quick knock. The one patient in his 20s, who expressed some concern over privacy, devised his own solution to turn the camera toward the wall temporarily while he showered.

### Care Team Use Factors Impacting Patient Acceptance

The clinical team’s use of the technology also impacted patient acceptance, specifically around patient orientation to technology, clinician etiquette regarding encounter introductions and use, and visual connection with each speaker. Patients described minimal or no orientation to the tablet. “They never told me about the iPad; I noticed when they came on-- ‘Hello hello hello’” [Patient 10]. Where an orientation was reported to take place, it was minimal: “They pointed it out to me and said…this is the [tablet] for nurses and doctors to call you” [Patient 11]. While this brief orientation would have been preferred, it did not bother most patients. A preference for an orientation was more prevalent among patients who did not have prior familiarity with using tablet devices or web conferencing technology.

Opportunities were also noted for improved etiquette around introducing each member of the team and positioning the tablet toward the speaker:

I have a bunch of different doctors on my care team…[it is] much harder to remember each one, [as I] don’t have visual clues. I see [them] only as 2D and honestly [there is an] unwillingness for everyone to get in front of the screen…one [clinician] is on the side, I hear their comments but I don’t know who that is…

Patient 3

This concern was not prevalent among all patients but may have improved acceptability among the few patients who voiced it.

## Discussion

### Principal Findings

Patients’ experience with telemedicine during an inpatient admission for COVID-19 was largely favorable. While most patients expressed a preference for in-person care where possible, telemedicine was an acceptable alternative given COVID-19 isolation precautions. Both nurses and physicians regularly used the technology to communicate with patients, thus validating past work [27] in which the balance of in-person versus telemedicine workflows was guided by acuity of clinical need. Telemedicine appeared to serve as a supplementary point of contact between in-person encounters, which patients primarily saw as a benefit to their overall experience and mental health. The technology reportedly played a meaningful role in mitigating the fear and anxiety associated with isolation precautions [31-37], though additional evaluation is needed to quantify this impact. Finally, with the exception of a desire by some patients for more hands-on examination and in-person interaction, patients felt their quality of care was no different as a result of inpatient telemedicine.

Direct and implied recommendations to improve inpatient telemedicine include increasing audio volume, allowing for a digital “knock” to serve as a warning prior to automatic turn-on, establishing an unobtrusive stable location for the tablet, adding a call-out button to the nursing team, increasing focus on physical touch, improving clinical team introductions, and standardizing patient orientation to the technology (Table 2).

These data are consistent with the past literature, which suggests that providers may overestimate their communication ability [41]. Given that a physician is required to evaluate each inpatient at least once per day, the frequency of physician encounters less than once per day reported by some patient suggests suboptimal communication and role confusion. A strong introduction includes the name, role, and responsibilities of each member of the care team, information that is often not shared even in nondigital clinical settings [42,43]. Training related to the importance of verbal introductions [44,45] and best practices to foster the patient–provider connection in the virtual setting may help mitigate the challenge of role confusion [46]. Clear badges or face cards [38,43,47,48] can also help, but these tools are not available in the digital setting. Instead, facial recognition technology is being explored to solve other problems in health care [49,50] and could be used to automatically display a virtual name badge.

Some patients were also concerned that aspects of their care may be missed owing to the lack of a hands-on examination. The importance of a hands-on physical examination—both for diagnostic and therapeutic purposes—is evidenced in the literature [51,52], but how to best adapt these learnings to patients are under isolation precautions remains an open question. Nurses were reported to use telemedicine more frequently than physicians, while also maintaining a frequent physical presence within the room, which is perhaps unsurprising given that nurses spend approximately 6-fold more time at the patient bedside than physicians in non–COVID-19 settings [53,54]. These COVID-19 data further validate past qualitative work, which suggests that nurses use telemedicine as a bridge to in-person care and “batch” care activities, such that physical assessment, medication delivery, meal delivery, and sanitation protocols all occur in a single room entry [27,55]. The impact of this shift, alongside the reported reduction in in-person physician assessments, on clinical outcomes is an area for future research.

To this end, inpatient telemedicine risks exacerbating pre-existing clinician reliance on technology over hands-on examination. Substitutes for an in-person examination, such as directing patients toward a self-examination with a digital stethoscope [56], pulse oximeter, or other technologies capable of remote transmission of data, even if appropriate from a diagnostic perspective [57], may be less acceptable to patients in the inpatient setting. Additional evaluation is therefore needed to determine the optimal ratio of in-person to virtual encounters.

**Table 2:** Implied technical and protocol recommendations to improve inpatient telemedicine in accordance with patient interviews.

Patient concern			Possible solutions
**Telemedicine technical setup**			
	Low audio volume	Improve the audio with more powerful provider-side microphone and patient-side speaker technology	
	Tablet position is suboptimally angled or too far from the patient	Stable, unobtrusive “home” for the tablet device within the patient room at an optimal distance and angle; standardized within all patient rooms with telemedicine capabilities	
	Tablet device automatically turns on without warning	Announcement of an incoming call with a digital “knock” and a visual and audible countdown prior to automatic web conference turn-on or “entry” into a room	
	Emergency situation where the patient wants immediate contact with the care team	Callout button direct to the nurse from the tablet device and the web conferencing system	
**Care team protocol when using telemedicine**			
	Desire for physical examination on a regular basis	Hands-on physical examination by the physician or nursing team with a dedicated stethoscope Exploration of patient self-exam using enabled devices such as stethoscope with remote transmission capabilities	
	Poor understanding of who is on the care team and their respective roles when using the web conferencing tool	Clinical team training emphasizing improved verbal introductions at each virtual encounter Automatic caption with the name or title based on facial recognition technology	
	Insufficient patient orientation to tablet use	Standard orientation to telemedicine, including self-directed exploration	

### Limitations

Insights from this evaluation are drawn from a small sample of patients within a single institution and therefore cannot be more broadly generalized. These exploratory interviews provide insight into an otherwise difficult-to-reach patient population with currently limited available data, though future work will benefit from expansion to diverse institutions and patient populations. Including patients who required intensive care may be particularly informative; safety and feasibility constraints limited our ability to capture these perspectives in this assessment. In addition, we purposefully sought a diverse set of voices in our sampling protocol, and our final sample overrepresented non-White participants on the basis of local demographics [58]. Increasing sample diversity in terms of languages spoken, given the predominance of English speakers in this sample, may benefit future work. Finally, these qualitative data may also complement and inform ongoing and future quantitative work that explores the impact of inpatient telemedicine on clinician workflows and, by extension, infection control and resource use [59].

### Conclusions

Inpatient telemedicine adopted for the purposes of infection control during the COVID-19 pandemic presents a novel use case of the technology, and our understanding of its impact on clinical workflows, patient outcomes, and the patient experience continues to evolve. This qualitative evaluation suggests that while patients still prefer in-person interactions in the hospital setting, inpatient telemedicine is broadly accepted given the need for COVID-19 isolation precautions. Perceived benefits include increased access to the clinical team and reduced anxiety, yet challenges around the technical setup, clinical team introductions, and physical examination remain. Further evaluation is needed to understand the impact on clinical outcomes and the optimal balance between in-person and virtual care in the hospital setting.

## Appendix

Multimedia Appendix 1 Semi-structured patient interview protocol.

## Abbreviations

PPE: personal protective equipment
