# Supplementary material for: Patient Perspectives of Inpatient Telemedicine During the COVID-19 Pandemic: Qualitative Assessment
Source: JMIR Form Res. 2022 Mar 30;6(3):e32933. doi: 10.2196/32933 (PMC8970158; doi:10.2196/32933)
Supplement: Multimedia Appendix 1 [file formative_v6i3e32933_app1.docx]

**Appendix A: Setting**

Individual telemedicine devices complemented pre-existing bedside telephones in which a patient could press a red button to call out to their nurse; at the time of analyses, patients were unable to call out from the tablet itself. All clinical team members including hospitalist and specialist physicians, nurses, respiratory therapists, trainees and others received instructions on system use and were encouraged to incorporate it into their clinical practice.

**Appendix B: Semi-structured Patient Interview Details and Protocol**

*Details*

Coordination with unit nurses facilitated the direct phone connection into the patient’s room at an appropriate time when the patient was awake and available to speak. Interviews were conducted between October 2020 through January 2021 and reflect the technical capabilities during this period. Interviews lasted approximately 30 minutes and took place primarily by phone. Early video interviews that took place via inpatient telemedicine gave the evaluation team direct experience with the platform itself and led to the decision the platform was best left available for clinical encounters; thus, the hospital bedside phone was the primary means of conducting the interview. Interview content focused on patient experience with the intervention, perceived impact on quality of care, clinical communication, and mental health and factors related to future use of the intervention.

*Protocol*

INTRODUCTION

1. What is your full name? What is your date of birth? [Team lead to capture in PHI-safe Box file]
2. How long have you been in the hospital? [How long ago where you discharged from the hospital?]
3. How have you used inpatient telemedicine during your hospital stay? [Adapt language to what patient uses, e.g. “Zoom” instead of “inpatient telemedicine”]

ACCEPTANCE & USE OF TECHNOLOGY

1. What was your experience with inpatient telemedicine like?
   1. What did you like about it?
   2. What did you not like about it?
2. When you are admitted to the M7 floor, what information did you receive about inpatient telemedicine?
   1. What information did you wish you had received?
3. How easily were you able to connect to inpatient telemedicine?
4. How often did the system automatically turn on during an incoming call?
5. How often did you call out to the nurse?
6. How often would your nurse come into your room while you were in the hospital? What aspects of care did he/she come in for?
   1. What about your physician?
   2. What about other health workers (e .g. respiratory specialists, physical therapy)?
7. What else do you use the technology (ipad) for?
8. Have you gone through any process to consent for a clinical study? How did that take place?

PERCEIVED QUALITY OF CARE

1. How do you think inpatient telemedicine has impacted the quality of your medical care?
2. How has inpatient telemedicine changed the way you talk with your doctor or nurse about your health?
3. Are some doctors or nurses better at using the inpatient telemedicine than others?
4. At the end of the day, how is your mood?
   1. How do you think inpatient telemedicine impacted it?
5. Under what circumstances would you NOT want to use inpatient telemedicine in the hospital? Are there any in which you would specifically WANT to use it?
6. If you were actively having trouble breathing, would you feel comfortable with the nurses responding via telemedicine, or would you prefer they physically come into the room?
7. If you had a magic wand, how would you change inpatient telemedicine to make it better?

FUTURE USE OF TELEMEDICINE

1. Have you had any experience before your stay at [health system] with video conference technology such as Zoom/Skype previously?
2. How do you plan to follow up with your doctor once you are discharged?
3. Do you have a way of connecting with your doctor by video once you are home?
4. What benefits or drawbacks do you see in using telemedicine once you are home?
5. Thinking about the future of this technology, are there certain kinds of patient in the hospital who might benefit from it?

CLOSING / DEMOGRAPHICS

1. Thank you so much for your time. Is there anything else you think we should know about inpatient telemedicine?
2. Just a few demographic questions before we wrap up: With which gender do you most identify?  ___Female ___Male ___Other (please specify) __________
3. Regarding your race and ethnicity, which do you identify with? You can pick as many as that apply: ___Latinx (Latino, Latina) ____American Indian or Alaska Native ___Asian ___Black or African American ____Native Hawaiian or Other Pacific Islander ____White ____Other________________

**Appendix C: Data Analysis**

Interviews were transcribed near-verbatim by a single designated researcher (EW) while other researchers with qualitative training conducted and observed the interviews (SV, SS, EA). For non-English speakers, a professional interpreter from the health system was enlisted to interpret interviewer questions and interviewee responses, which were then transcribed in English for analysis. A physician health services researcher (SV) supervised interview transcript analysis (DP, MS, JL) using a rapid analytic process structured around the *a priori* categories described above.

Following code and theme finalization, hypothesis-driven sub-analyses were conducted to understand how variation in age, language, race/ethnicity, and past experience with web-conferencing technology impacted patient perspectives. These sub-analyses are presented alongside key themes where relevant.

**Appendix D:** **Demographics of Patient Participants Hospitalized with COVID-19**

| **Demographics** | **n (%)** |
| --- | --- |
| **Sex** |  |
| Males | 11 (55%) |
| Females | 9 (45%) |
| **Age (Decades)** |  |
| 20s | 1 (5%) |
| 30s | 1 (5%) |
| 40s | 1 (5%) |
| 50s | 6 (30%) |
| 60s | 9 (45%) |
| 70s | 1 (5%) |
| 80s | 1 (5%) |
| **Race/Ethnicity** |  |
| White | 6 (30%) |
| Latino | 6 (30%) |
| Asian/Indian/Pacific Islander | 6 (30%) |
| Black/African American | 2 (10%) |
| **Language Spoken** |  |
| English | 16 (80%) |
| Spanish | 3 (15%) |
| Tongan | 1 (5%) |
| **Total Patients** | **20 (100%)** |
